# Effects of Ankle Foot Orthoses on the Gait Patterns in Children with Spastic Bilateral Cerebral Palsy: A Scoping Review

**DOI:** 10.3390/children8100903

**Published:** 2021-10-10

**Authors:** Diogo Ricardo, Maria Raquel Raposo, Eduardo Brazete Cruz, Raul Oliveira, Filomena Carnide, António Prieto Veloso, Filipa João

**Affiliations:** 1 CIPER, Faculdade de Motricidade Humana, Universidade de Lisboa, Estrada da Costa, 1499-002 Cruz-Quebrada—Dafundo, 1499-002 Oeiras, Portugal; mraquelbraposo@gmail.com (M.R.R.); eduardo.cruz@ess.ips.pt (E.B.C.); roliveira@fmh.ulisboa.pt (R.O.); fcarnide@fmh.ulisboa.pt (F.C.); apveloso@fmh.ulisboa.pt (A.P.V.); filipajoao@fmh.ulisboa.pt (F.J.); 2 Escola Superior de Tecnologia da Saúde de Lisboa (ESTeSL), Instituto Politécnico de Lisboa, Av. D. João II, 1990-096 Lisboa, Portugal; 3 Escola Superior de Saúde, Instituto Politécnico de Setúbal, Campus do IPS-Estefanilha, 2910-761 Setúbal, Portugal

**Keywords:** child, cerebral palsy, gait analysis, orthotic devices, biomechanics

## Abstract

Background: Cerebral palsy (CP) is the most common cause of motor disability in children and can cause severe gait deviations. The sagittal gait patterns classification for children with bilateral CP is an important guideline for the planning of the rehabilitation process. Ankle foot orthoses should improve the biomechanical parameters of pathological gait in the sagittal plane. Methods: A systematic search of the literature was conducted to identify randomized controlled trials (RCT) and controlled clinical trials (CCT) which measured the effect of ankle foot orthoses (AFO) on the gait of children with spastic bilateral CP, with kinetic, kinematic, and functional outcomes. Five databases (Pubmed, Scopus, ISI Web of SCIENCE, SciELO, and Cochrane Library) were searched before February 2020. The PEDro Score was used to assess the methodological quality of the selected studies and alignment with the Cochrane approach was also reviewed. Prospero registration number: CRD42018102670. Results: We included 10 studies considering a total of 285 children with spastic bilateral CP. None of the studies had a PEDro score below 4/10, including five RCTs. We identified five different types of AFO (solid; dynamic; hinged; ground reaction; posterior leaf spring) used across all studies. Only two studies referred to a classification for gait patterns. Across the different outcomes, significant differences were found in walking speed, stride length and cadence, range of motion, ground force reaction and joint moments, as well as functional scores, while wearing AFO. Conclusions: Overall, the use of AFO in children with spastic bilateral CP minimizes the impact of pathological gait, consistently improving some kinematic, kinetic, and spatial-temporal parameters, and making their gait closer to that of typically developing children. Creating a standardized protocol for future studies involving AFO would facilitate the reporting of new scientific data and help clinicians use their clinical reasoning skills to recommend the best AFO for their patients.

## 1. Introduction

Cerebral palsy (CP) is the most common cause of motor disability in children [1,2,3]. Overall prevalence of CP is around 1 per 500 live births worldwide [2,3,4,5]. CP is a complex pathology that describes a group of impairments and motor disorders [5] with different presentations and functional levels [6].

The gait deviations that occur in children with CP are among other factors, due to inadequate muscle action [7]. Instrumented clinical gait analysis has been a great tool for planning intervention and assessing outcomes in the rehabilitation process of children with CP [2,8]. However, the use of all the outcomes within the three-dimensional kinematics or kinetics data to support classifying gait patterns in CP is still scarce [8], due to the almost exclusive use of the sagittal plane kinematic outcome in the majority of the gait classification systems [9,10].

Among several gait classification systems in children with CP, and particularly in bilateral spastic CP, Rodda et al. [11] identified several gait patterns and reported a high intra-rater reliability and moderate inter-rater reliability [9]. More recently Papageorgiou et al. [10] concluded that the characteristics presented by Rodda were considered as the most exhaustive ones, always including information about the co-occurring deviations across all lower limb joints [10].

This classification is based on clinical insight and biomechanical principles, and identifies five basic patterns of sagittal plane gait in spastic bilateral CP, namely true equinus, jump gait, apparent equinus, crouch gait, and asymmetric gait. These definitions are intended to be starting points for the guidelines for the planning of the rehabilitation process of children with CP. This allows not only the assessment of the most suitable orthosis for each case but also other surgical and non-surgical interventions, helping in the clinical decision-making process [11].

The use of ankle foot orthoses (AFO) is commonly prescribed to prevent the development or progression of deformity, and to control motion to improve dynamic efficiency of the child’s gait [12,13]. There is a wide selection of AFO that can be used in the rehabilitation processes. However, their intended function depends mainly on their configurations, the material used, and its stiffness. Any alteration of these three components will alter the control that the AFO has on the patient’s gait [14]. There are multiple designs, either fabricated as a one-piece of thicker thermoplastic AFO that restricts ankle and foot motion in all three planes (SAFO), or a flexible and dynamic AFO that allows some degree of sagittal plane motion (DAFO); a one piece design with a posterior malleolar trim line (posterior leaf spring-PLS), a two-piece design with a hinged joint that typically allows for dorsiflexion (HAFO), or a one piece anterior shelf design that promotes knee extension (GRAFO) [15,16,17].

Overall, studies involving gait and kinematic analysis have indicated that pathological gait in the sagittal plane can be improved using AFO [2,18,19], however it is not consensual about what factors are improved and how they have been improved. Thus, an assessment of the biomechanical characteristics and functional ability of the participants at baseline is crucial to track existing changes during the use of AFO [20]. Many studies involving orthotic use with CP patients present a wide variety of discrepancies in inclusion criteria or baseline assessments; missing information about orthosis design and construction, and how they are used; and different types of outcomes that can bias the indicated results. Previous systematic reviews have not focused on specific CP subgroups or referred to gait pattern classifications, thereby including a wide range of gait abnormalities, or have included the information of lower quality studies [21,22,23,24].

Due to the broad specter of physical presentations of children with CP, the aim of this review is to determine the effects of different types of ankle foot orthoses on the gait of children with spastic bilateral CP, presenting specific recommendations for this particular subset, and whenever possible refer to its effects on the five different sagittal gait patterns [11].

## 2. Materials and Methods

### 2.1. Search Strategy

A preliminary search was performed to select keywords related to the population, intervention, and outcomes using the PICO framework [25]. The keywords selected from the MeSH database in MEDLINE were: cerebral palsy, child, adolescent, orthotic devices, foot orthoses, splints, gait, kinematics, kinetics, walking, hip, hip joint, knee, knee joint, ankle, ankle joint, articular range of motion, walking speed, and International Classification of Functioning, Disability, and Health (ICF). Subsequent refinement searches were performed to obtain results. The selected keywords were joined by the words “AND” and “OR”. The search equation was adapted according to the database where it was applied (Table A1). The search was performed between January and July 2018, and included all records from the onset of each database. A secondary search was conducted in February 2020 with no other studies meeting the eligibility criteria. A keyword search was performed to match words in (all fields) the title, abstract, or keyword fields. The publication date was not restricted. Whenever possible filters on language were applied (Portuguese and English) (Appendix A).

The search to identify the relevant articles for this review was carried out in the following databases: Pubmed, Scopus, ISI Web of Science, Cochrane Library, and Scielo. To identify potentially relevant trials that were unpublished or ongoing, a search was also performed in the database of the World Health Organization International Clinical Trials Registry Platform (WHO ICTRP) and in the US National Institutes of Health (ClinicalTrials.gov).

### 2.2. Selection Criteria

#### 2.2.1. Eligibility Criteria

The methodology used for this review followed the Cochrane guidelines [26]. The eligibility criteria for the selected articles were randomized clinical trials (RCT) and controlled clinical trials (CCT) (study design); written in English, Portuguese, or Spanish (language); with a focus on the pediatric population with bilateral CP (population) that used an AFO as a therapeutic intervention (intervention). The exclusion criteria were the use of functional electrical stimulation or robotic assisted therapy, and the existence of previous surgical or medical procedures (intervention). The outcome measures considered were the biomechanical gait parameters and/or functional abilities, including spatial-temporal, kinematic, kinetic, and gross motor function outcomes (outcomes).

#### 2.2.2. Study Selection

The article selection was conducted by two independent reviewers (D.R. and M.R.R.), after duplicate removal and checking the articles’ titles and abstracts against the eligibility criteria. The full text of the remaining articles was read. A bibliographic reference software manager (Mendeley V. 1.19.3) was used to assist the selection process. Whenever the two main investigators could not reach a consensus, a third external reviewer (E.B.C.) would intervene.

### 2.3. Methodological Quality (Risk of Bias)

The assessment of the quality of the included studies was the PEDro Risk of Bias Tool [27,28], for a minimum score of ≥5 points, which usually represents an adequate methodological quality study [29]. The rating of the studies and scoring of their methodological consistency were conducted by two reviewers (D.R. and M.R.R.), and, in case of disagreement or any discrepancies in scores, details were discussed with a third reviewer (E.B.C.). Furthermore, alignment between the PEDro scores and the Cochrane approach was verified for a broader assessment of the quality of the included studies [29].

### 2.4. Data Extraction

The characteristics of each selected study were extracted to compare the features across the studies. Author names, date of publication, study type and design, population characteristics and eligibility criteria, sample size, intervention type and duration, variables, measure instruments, and main findings were included.

## 3. Results

### 3.1. Article Selection

**Figure 1:**
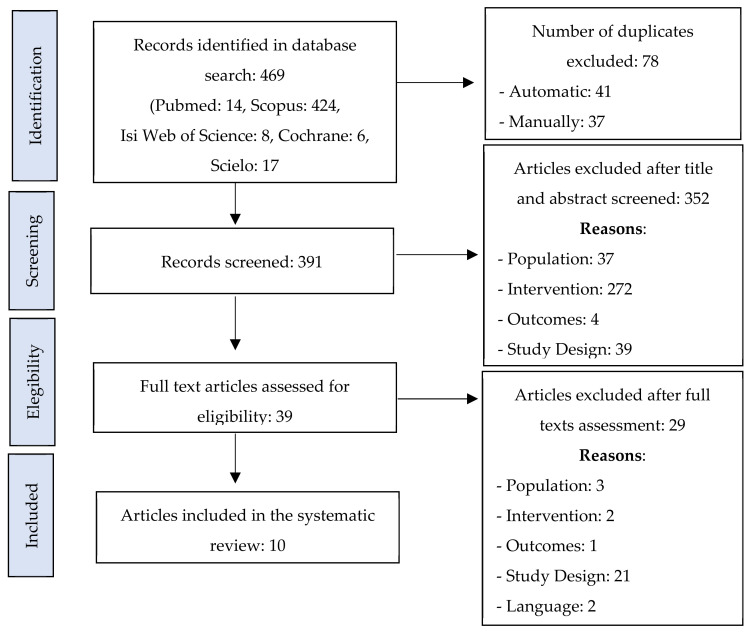
Flowchart of the article selection process.

The initial search strategy identified 469 articles. After 78 duplicates were excluded, a further screening based on the title and abstract to assess the relevance of the articles excluded 352 articles. These articles did not meet the criteria of population (37), intervention (272), outcomes (4), and study design (39). A full text reading excluded 29 articles based on the criteria of population (3), intervention (2), outcomes (1), study design (21), and language (2). This resulted in a total of 10 articles that met our inclusion criteria and were included in our review flowchart (Figure 1).

### 3.2. Article Characteristics

The selected articles were published between 1997 and 2016. Of the 10 studies that were included, five were RCT [15,30,31,32,33] (three with a crossover design) and five were CCT [34,35,36,37,38]. The duration of the studies ranged from 1 day to 12 months in total. All studies compared at least one type of AFO intervention with barefoot, shoes, or other types of AFO interventions. The range of measurement instruments that were used included: optoeletronic systems, ankle accelerometer, force plates, and the Gross Motor Function Measure (GMFM) tool. The studies reported spatial-temporal parameters (walking speed, stride length, and cadence), kinematic outcomes (range of motion), kinetic outcomes (ground reaction force, joint moments, and joint power), and functional outcomes (GMFM). This enabled the compilation of data detailed in Table 1.

The studies with fair to strong methodological quality were as follows: six studies with 4–5/10, one study with 6/10, and three studies with 8/10 on the PEDro scale (Table 2). All articles specified their “eligibility criteria”, “follow-up”, “intention to treat”, and “statistical comparison”. The “blind distribution”, “blind subject”, “blind therapist”, and “blind assessor” were the items most often not verified. Three studies [15,30,31] managed to create blind assessment conditions, only two studies [15,30] had “blind distribution”, and only one study [31] had unknowing therapist. No studies had “blind subjects”, as it is not possible to use AFO without knowing it. Three studies [34,35,38] did not have equal circumstances at baseline (“similar prognosis”) for their groups, as they used typically developed children for control group.

#### 3.2.1. Characteristics of the Participants (Sagittal Gait Patterns)

Across all studies, there was a total of 347 participants (289 children with CP and 58 typically developing children [34,35,38]). Most studies included only children with spastic bilateral CP (285). Despite this, one study [37] presented a heterogeneous population, with four children with spastic unilateral CP. However, as the results were presented separately, we did not include them in this review.

Only a small percentage of the total participants had their gait patterns identified. Two studies referred to the sagittal gait patterns classification [32,38], identifying in total 18 participants with jump gait pattern, 5 true equinus, and 3 crouch gait pattern.

#### 3.2.2. Types of AFO

The majority of interventions were centered in the comparison of gait when using ankle-foot orthosis and when walking barefoot [15,33,34,35,36,37], or using conventional shoes [31,32,38]. The type of AFO is central in most studies [15,30,33,34,35,36,37,38], but information about AFO construction, design and materials, as well as overall lower limb alignment and footwear are partially missing in every study.

We identified five different types of orthoses: 178 participants used solid ankle foot orthoses (SAFO) [30,32,33,34,35,36,37], 57 participants used dynamic ankle foot orthoses (DAFO) [31,35,37,38], 24 participants used posterior leaf spring (PLS) [33,34], 46 participants used hinged ankle foot orthoses (HAFO) [33,36,38], and 19 participants used ground reaction ankle foot orthoses (GRAFO) [15]. We found that overall, studies had no clear and consensual definition of the different types of AFO, and there was more than one description and configuration for the same terminology. In some of the studies, participants wore more than one type of orthoses [33,35,36,37,38], and in other studies some participants did not use any type of AFO [15].

#### 3.2.3. Type of Outcomes

The main outcomes that were found were the following: spatial-temporal parameters [15,33,35,36,37,38], range of motion (RoM) [33,35,36,37,38], ground reaction forces [35], joint moments [33,35,36,38], and joint power [33,35,36,38]. Secondarily, some studies presented functional parameters, isolated or correlated with the biomechanical analysis [38]. The most frequently used tool was the Gross Motor Function Measure scale (GMFM) [30,31,32,33].

Most articles did not directly relate the reported outcomes with changes in the gait pattern in children with CP. Still, whenever possible, outcomes observed in the sagittal plane were associated with changes in the gait pattern.

##### Spatial-Temporal Parameters

One study compared gait in children with CP barefoot at baseline and after 4 weeks of DAFO or HAFO wear, and found significant differences (*p* ≤ 0.006) across all measured spatial-temporal parameters (walking speed, stride length, and cadence) [38]. In studies that compared either children with CP wearing AFO with their typically developed peers or children with CP wearing AFO and barefoot, it was shown that use of AFO (regardless of the type) had a significant increase or an approximation to normal reference parameters in walking speed [15,38], step [33] and stride length [15,33,35,36,37,38], and a significant decrease towards normal cadence [15,33,37,38].

Nevertheless, there were studies that reported no significant differences for walking speed [33,35,36,37], nor significant differences for cadence [33,35,36] irrespective of AFO type or study design.

##### Kinematic Outcomes

The most often used kinematic parameter was RoM of the lower limb joints. For instance, significant improvement towards dorsiflexion of the ankle at the initial contact, and swing phase was observed [33,35,36,37,38], but, because the orthoses limit the plantar flexion, there was a significant decrease in RoM in the push-off stage of the pre-swing phase [35]. Maximal dorsiflexion in stance phase improved significantly with the use of SAFO [33,35,36]. It was also reported that the HAFO can produce excessive dorsiflexion during the stance phase [36].

While the most significant changes when wearing AFO are in the ankle RoM, in the knee RoM some differences were found, particularly in knee flexion on initial contact when compared to the barefoot condition [35,38]. Furthermore, children with CP wearing AFO showed a significantly greater range of motion of the shank [34]. No significant difference in knee RoM was found between the different types of AFO [33,35].

One study showed that children wearing DAFO were found to have a significantly greater hip flexion at initial contact [35], but overall, most studies found no significant changes at the hip joint, regardless the type of AFO [33,36,37,38].

##### Kinetic Outcomes

Only four studies reported kinetic parameters. One study reported that when using a SAFO or DAFO, there was a significant increase in the ground reaction force at the push-off when compared with the barefoot condition in children with CP [35]. An increase in the maximum plantar flexion moment in the terminal stance (push-off) was also reported, regardless of the type of AFO, with results similar to those of healthy children [33,35,36,38]. Peak knee extensor moment in early stance was significantly increased in the HAFO configuration compared with barefoot condition [33].

Regarding joint power, no significant difference was found in any of the analyzed joints between barefoot condition and AFO condition [33,35,38]. However, it was also reported that the peak of ankle power (that occurs at the push-off phase) when wearing a HAFO was similar to the barefoot condition [36], and between the configurations, the SAFO decreased peak power generation in stance significantly more than the PLS [33].

##### Functional Outcomes

To complement the biomechanical data, we were also interested in functional outcomes that the CP children may have reported with the use of AFO. The GMFM was the most often used tool, and studies showed it is responsive to change and can be used to evaluate the progress of a child while wearing AFO [39]. Although some of the included studies presented poor biomechanical data, they used this measure to evaluate the progress of AFO use in rehabilitation [30,31,33]. Most of the studies showed that the percentage scores for this scale were significantly higher when the patients wore the AFO [30,31,32], with the exception of one study where the AFO use did not significantly improve skills within the standing dimension of the GMFM [33]. The changes in some dimensions and total score of GMFM were also significantly higher for independent walkers compared to children with CP using assistive devices while wearing DAFO [31].

## 4. Discussion

The main focus of this review was to assess the effects of AFO on gait in children with spastic bilateral CP, with particular attention to effects on different sagittal gait patterns. Identifying the gait type is useful in guiding orthotic options [40], and its use, coupled with the three-dimensional gait analysis, has been helpful in the clinical decision-making process. As a result, we have selected sagittal gait pattern classification [11] to help gather and systematize information. However, very few studies referred to such classification, making it difficult to summarize the data in the way planned in the protocol.

Fundamentally, clinical gait analysis for children with bilateral CP is very complex, since bilateral impairment of the lower limbs is often met with different sagittal gait patterns in each limb, sometimes even overlapping due to multiple gait abnormalities.

The lack of gait pattern classification makes it more difficult to determine the mechanical and functional AFO characteristics needed to improve the different gait phases and overall performance. Two studies [32,38] did use the sagittal gait patterns [11] to identify and categorize clinical subsets, although only one [38] provided the participants with the type of AFO indicated in the classification.

The appropriate AFO prescription is a practice that requires the clinician to perform a thorough physical examination and observational gait analysis, regardless of the age or Gross Motor Function Classification System (GMFCS) level of the child with CP [40]. Although consistent guidelines are lacking in this field [41], when applying an AFO, the aim is to correct and stabilize the biomechanical alignment of the foot and ankle, prevent the appearance or worsening of a musculoskeletal deformity, maintain the outcome of a surgical procedure, and ultimately improve gait [13].

The rationale behind the selection of each AFO and its prescription is missing in most studies. One study used the GMFCS to select the AFO to be used [34]; one study used the AFO already owned by the children with CP but without describing criteria [32]; two used the results of similar studies made previously [31,36]; one study made their own recommendations after a clinical and biomechanical assessment [37]; and three studies did not declare the criteria followed [30,35,37].

Nevertheless, results suggest that overall, AFO use may positively impact the gait of children with spastic bilateral CP. Spatial-temporal parameters, such as walking speed and stride length, reveal an approximation to normal reference [34,35,36,37], suggesting a better gait efficiency and probably less energy expenditure [33].

Overall, children with CP wearing any type of AFO presented significant differences in the range of motion of the ankle, when compared to the barefoot condition. Regardless of the AFO type, its use appears to reduce pathological plantarflexion, common in several of the bilateral CP gait patterns [35]. However, some types of orthoses (DAFO, SAFO, and GRAFO) are particularly more effective in controlling tibial progression and consequently promote knee extension during stance [32]. This can impact and modify the crouch gait pattern of CP children, approximating it to that of healthy subjects.

In children with spastic bilateral CP, there were significant increases in ground-reaction force and joint moments at push-off while wearing different AFO [35]. This demonstrates that up to 5 degrees of dorsiflexion of the ankle inside the AFO is more advantageous and induces an optimal muscle length in the calf muscles, approximating the plantar flexion moment to that of normal values [35,37].

Of the ten studies included in this review, only three focused on functional gains, and only one of the studies presented both biomechanical and functional data. Functional assessments are widely use in the rehabilitation of children with CP and should be more often correlated with biomechanical variables.

### Methodological Considerations of This Review

We identified methodological limitations that are common in this type of study. Due to our eligibility criteria, the number of articles included was lower than other similar reviews. Of the 10 studies included, there was no common primary outcome between them. Although biomechanical and/or functional outcomes were found in all studies, the study designs are vastly heterogeneous (different samples sizes; wide range of age of participants; typically developed children control group versus children with CP barefoot control group; one day studies versus 12 months follow-up). This limits our ability to compare results due to the wider confidence intervals and a lower precision of the outcome measurements [42]. The point of statistical significance may be misleading, and this analysis may be leaving out some rehabilitation issues.

In CP research, CCT compares changes between groups to evaluate the efficacy of any treatment, but usually they lack reliable measures to detect changes that occur, which may be important from a clinical point of view [43]. In evidence-based medicine, the RCT is the highest level of evidence to be provided [44], and is the design of choice when comparing two or more healthcare interventions [29,44]. However, randomization may sometimes be affected by the number of participants, number of comparison groups, duration of the protocol, and the overall study design when studying AFO intervention. This may be a challenge because of differing clinical gait presentations and AFO requirements, thus we found that CCT are the more common for this population. The concealment of the allocation from parents and healthcare teams is a problem that practically limits this type of research [45,46].

Most studies included in this review were long-term follow-up studies [15,30,32,33,36,37,38] investigating the effects of the AFO for more than four weeks [47]. Studies with longer follow-up periods have also accounted for two weeks of rest between different orthosis [36,37]. This is relevant, as there were trials with a crossover design, where more than one type of orthosis was tested on the same day, raising concerns about the issue of carry-over effect between the different orthosis [31,32]. We suggest that future studies account for a proper wash-out period between trials [48].

Few authors advocate for an acclimatization period to ensure that the gait pattern is completely adapted to the altered ankle function as induced by the prescribed AFO, which may have impacted the results of their study [49]. Three studies allowed the children to wear the AFO one to three months prior to the first gait assessment so that the participants could gradually adapt to wearing them for the entire test day [33,36,37,38]. In two studies, children were already wearing their currently prescribed AFO [31,34]. Only one study reported the number of hours per/day/week that the subjects wore their AFO, but in all others that information was missing [15].

There are a wide variety of AFOs used in clinical practice, which are characterized by their design, the material used, and the stiffness of that material [14]. We have encountered at least five different types of AFO, but their definition was not always clear. The lack of nomenclature standardization also makes communication between researchers difficult [50].

Only one study used a prefabricated standard AFO [32], and in the remaining custom-made AFO were assigned for each participant [15,30,33,35,36,37,38]. Recent studies suggest that the initial outcomes are the immediate biomechanical response to the effect to the physical constraint imposed by the standard AFO, particularly the AFO stiffness [19,49]. On the other hand, custom-made AFO can be optimized with fine adjustments to its design and/or to the footwear prescription, in order to focus on optimal stiffness and increase its effects on gait pattern [14,51].

Even though an AFO is a frequently prescribed intervention for children with CP, rigorous evidence of their efficacy is limited [52], mainly because of the heterogeneity of outcome measures among researchers, which limits comparison between studies [53]. Although previous reviews have reported similar results and identified some of the limitations described above, still none have reported consistent guidelines for future studies [10,21,22,23,24]. Particularly, the absence of information about the clinical reasoning behind the AFO prescription, the selection of AFO design and construction, materials (including stiffness and thickness), AFO/footwear combinations, tuning, and acclimatization periods, makes it difficult to compare results within studies [50,54]. For instance, Kerkum et al. [47] reported that ankle ROM was significantly less reduced by both stiff and flexible spring-hinged AFO, and there was also a reduction in the ankle power when using a more rigid AFO. In this study, the authors used an instrument to measure the mechanical properties of the AFO and reported all the parametrization that was used for the AFO design. The differences found in gait kinematics and kinetics due to the stiffness of the AFO are only possible to compare with studies that also report the mechanical characteristics of the AFO, and that seems to be one of the greatest flaws in research regarding this topic [50].

Generically, the gait analysis protocols are not standard and have systematic errors related to extrinsic and intrinsic factors [55]. Regarding the use of 3D gait analysis in children with CP, several reliability studies identified that in the barefoot condition, kinematic and kinetic variables present with deviation between sessions, due to number of gait trials [56], biomechanical models and marker setup [57], or gait patterns and affected sides [58,59]. In turn, many studies report difficulties in 3D motion analyses when children with CP are wearing an AFO (especially when modeling ankle kinematics). When assessing the gait of children with CP wearing AFO, the marker setup usually sits on the surface of the AFO and shoe, making the assumption that they are the same rigid segment [60]. This may cause the interaction shank/ankle/AFO to present with some deviations. Ries et al. [16] attempted to minimize the influence of the AFO on shank and ankle kinematics by placing technical markers in a way that they were not to be covered or moved when the AFO was worn. By measuring the angle between the plantar surface of the shoe and the tibia, this study presented an alternative of measuring the true ankle position or the true neutral angle of the AFO.

Even though some methodological limitations are well reported, studies involving 3D gait analysis with the use of AFO should implement processes to minimize the error associated with their protocols, and state what measures they have included to assure that the outcomes of their research singles out the AFO effect.

It is also important to use tools such as the International Classification of Functioning, Disability, and Health (ICF) to standardize the report of results within the health-related domains [61]. Currently, there are specific ICF core sets for CP patients, therefore future studies should summarize the outcomes in this framework and create a common language across healthcare professionals [62].

Overall, we considered that there is need to standardize the AFO research, which can optimize the biomechanical properties and simplify future studies, making it possible to replicate results and provide better options for children with CP and their families [50].

## 5. Conclusions

In this review, we found that AFO use seems to have an immediate and a long-term effect in improving the sagittal gait patterns in children with spastic bilateral CP. However, most studies included heterogeneous groups with different gait patterns, and there were different approaches to the use of AFO. There is a need for future studies to invest in higher methodological quality protocols.

We propose the creation of a standardized protocol for future studies involving AFO and children with CP. There is a need to develop consistent AFO prescription algorithms that are designed specifically for each gait pattern. It should also include information about periods for AFO acclimatization and the need for fine tuning, appropriate follow-up periods to ensure full effect of AFO, appropriate wash-out periods, reports on hours per day of AFO usage, and AFO design, materials, and construction. This would facilitate the report and replication of new scientific data and help clinicians use their clinical reasoning skills to recommend the best AFO for their patients.

The rationale for these options needs to be more objective and evidence-based, which in the future may represent both improved assessment tools as well as a more effective therapeutic intervention.

## Figures and Tables

**Table 1:** Participants, sample details, methods, and main results.

Authors	Year	Study Design	Population Characteristics	Eligibility Criteria	N	Duration	Intervention/Procedure	Variables	Measurement Instruments	Main Results and Author’s Conclusions
Bjornson, 2006 [31]	2006	Randomized crossover controlled trial	23 children with spastic CP (age: 4.3 ± 1.5 years)	Children with spastic diplegia CP, 12 to 96 months, GMFCS I to III, bilateral use of AFO with free plantarflexion.	23	1 day	DAFO and shoes. GMFM used once with/without the orthoses during a same day evaluation.	Functional skills (GMFM scores).	GMFM.	The GMFM percentage scores for all dimensions were significantly higher with the patients wearing the DAFO (*p* ˂ 0.001). There seems to be a non-significant negative correlation of age to standing skills change, suggesting that DAFO effect may decrease with age, up to the age of approximately 7 years (*p* ˂ 0.001).
Bjornson, 2016 [32]	2016	Randomized crossover controlled trial	11 children with spastic CP (age: 4.3 ± 1.04 years)	Children with spastic diplegia CP; GMFCS I to III; bilateral use of AFO > 8 h/day, >1 month.	11	4 weeks (2 weeks without AFO and 2 weeks with AFO)	SAFO and shoes. Community based walking with/without AFO with a multiaxis accelerometer.	Functional skills (average total strides per day; % daytime hours walking; average number strides >30 strides/min; peak activity index).	StepWatch (Ankle accelerometer).	No significant difference was found in the primary outcome of average daily total step count between AFO-ON and AFO-OFF (*p* = 0.48). AFO did not improve walking activity levels.
Buckon, 2004 [33]	2004	Randomized crossover controlled trial	16 children with spastic CP (age: 8.3 ± 2.3 years)	Children with spastic diplegia CP; GMFCS I to II; bilateral use of AFO, 6 to 12 h daily >3 month.	16	1 year (a baseline assessment after three months of no AFO wear, and an assessment at the end of each AFO three-month wearing period	Barefoot or HAFO or PLS or SAFO.	Functional skills (GMFM scores); gait analysis data (kinematic variables at the pelvis, hip, knee, and ankle; Kinetic variables at the hip, knee, and ankle; Velocity, stride length, step length, and cadence).	Optoelectronic system; force plates; GMFM.	AFO use, regardless of configuration, did not significantly alter pelvic and hip kinematics and/or kinetics from the barefoot condition. At the knee there was no significant kinematic change. All AFO configurations significantly altered ankle kinematics during the stance and swing phases of gait: dorsiflexion at initial contact (*p* = 0.0001), peak dorsiflexion in stance (*p* < 0.009), timing of peak dorsiflexion in stance (*p* < 0.003), peak dorsiflexion in swing (*p* < 0.0002), and dynamic ankle range (*p* < 0.0001) compared with barefoot. Between the configurations, peak dorsiflexion in stance was significantly greater in the HAFO than the SAFO (*p* = 0.01), and the timing of peak dorsiflexion in stance was significantly later in the stance phase in the HAFO compared with the SAFO (*p* = 0.005). In conjunction with the changes in ankle kinematics, ankle kinetics (peak dorsiflexion moment in early stance [*p* = 0.0001], peak plantarflexion moment in early stance [*p* = 0.0001], peak power generation in stance [*p* < 0.008], and the timing of peak power generation [*p* < 0.005]) changed significantly in all the AFO configurations compared with barefoot. All of the AFO configurations significantly increased step (*p* < 0.005) and stride length (*p* < 0.006) compared with barefoot, while significantly decreasing cadence (*p* < 0.0005). Therefore, velocity did not increase significantly with AFO use compared with barefoot. Velocity was significantly slower in the HAFO compared with the PLS (*p* = 0.009), owing to a 17% decrease in cadence in the HAFO, an 11% decrease in the PLS, and a 13% decrease in the SAFO, compared with barefoot. AFO use did not significantly improve skills within the standing dimension of the GMFM. However, all AFO configurations significantly improved skills within the W/R/J dimension compared with the barefoot condition (*p* < 0.002).
Degelean, 2012 [34]	2012	Non-randomized controlled clinical trial plus healthy controls (repeated measures design)	20 children with spastic diplegic CP (mean age: 7.6 ± 1.7 years) + 20 typically developing children (mean age: 7.8; ± 1.4 years)	Children with CP of the spastic diplegia type within the age of 4 and 12 years; no history of orthopedic surgery; no botulinum toxin injections within the last year; GMFCS level I or II; use of posterior leaf spring-type or solid AFO either in habitual walking or during physical therapy sessions.	20 + 20	1 day	Spring AFO or SAFO vs. barefoot. Participants walked at a comfortable speed on an 8 m walkway with AFO and barefoot. The task was recorded using an optoelectronic system detecting passive retro-reflective markers.	Gait analysis data (trunk movements; angular velocities; peak-to-peak excursions in trunk angular displacements; elevation angles of the thigh, shank, and foot).	Optoeletronic system.	Children with CP showed greater trunk sway excursion and angular velocity in both the sagittal and frontal directions compared to the control group (*p* ˂ 0.05). Children with CP have greater sagittal and frontal trunk movements compared to typically developing children, but the difference in frontal motion was higher than in sagittal motion (*p* ˂ 0.05). The use of any of AFO improved lower limb intersegmental coordination during gait in children with spastic diplegia by making it closer to a typical, mature gait pattern (*p* ˂ 0.05). This was indicated by a significant greater ROM of the shank and a decreased ROM foot. However, wearing AFO results in increased trunk motion, which may be problematic in the context of difficult postural control.
El-Kafy, 2014 [15]	2014	Randomized parallel group controlled trial	57 children with spastic diplegic (mean age: 7.3 ± 1.3 years)	Children with CP of the spastic diplegia type within the age of 6–8 years old; under 40 kg; cognitively able to understand simple instructions; no recurrent medical issues; no allergic reactions to the adhesive tape or any other materials; no visual, auditory, or perceptual deficits or seizures; no previously use of TheraTogs orthotic undergarment, or strapping system and ground reaction ankle foot orthosis; no botulinum toxin in the lower extremity musculature during the past 6 months or other spasticity medication within 3 months of pre-treatment testing.	19 + 19 + 19	2 h/day, 5 days/week for a total of 12 weeks	Control group (A)—traditional neuro- developmental physical therapy. Study group (B)—A + TheraTogsTM orthotic undergarment and strapping system for both lower extremities. Study group (C)—B + received GRAFO in both lower limbs. Participants walked at a comfortable speed on an 8 m walkway with AFO and barefoot. The task was recorded using an optoelectronic system detecting passive retro-reflective markers.	Gait analysis data (gait speed; cadence; stride length; hip and knee flexion angles).	Optoeletronic system.	There were significant differences among the 3 groups pre-treatment in all measured variables (gait speed, cadence, stride length, and bilateral hip and knee flexion angles), and that they were present post-treatment (*p* ˂ 0.05). This is due to the improvement of the plantar flexion, knee extension coupling, and knee and hip extension angle in mid stance provided by the GRAFO. The statistically significant differences post-treatment, in all parameters, were greater in group C than that in both groups A and B (*p* ˂ 0.05). The results concerning the mean values of bilateral hip and knee rotational angles between both groups B and C revealed that there were no statistically significant differences in either pre- or post-treatment evaluation times (*p* ˂ 0.05).
Lam, 2005 [35]	2005	Non-randomized controlled clinical trial plus healthy controls (repeated measures design)	7 boys and 6 girls with spastic diplegic CP (mean age: 5.9 ± 1.81 years) + 18 typically developing children (age matched)	Spastic diplegia CP with mainly moderate dynamic equinus (modified Ashworth scale 1–3); no significant coronal or rotational deformities; no botulinum toxin injections within the preceding 5 months; good vision; the ability to comprehend instructions; be able to walk independently.	13 + 18	1 day	AFO and DAFO. Barefoot (healthy subjects control group).	Gait analysis data (stride length; stride time; speed; stance time; swing time; stance/swing ratio; cadence; range of motion parameters; moment parameters; power parameters).	Optoeletronic system; force platform.	CP patients had significantly shorter stride length than normal. Both AFO and DAFO conditions significantly increased stride length (*p* ˂ 0.05). The mean stride length in CP patients walking barefoot (0.69 ± 0.14) was 65% of the healthy age matched children. The stride length was significantly increased when the subjects were wearing AFO (0.74 ± 0.15) or DAFO (0.81 ± 0.15). Concerning the total ROM, there was a reduction in range of motion at the ankle joint between the barefoot (22.39 ± 6.78), AFO (12.44 ± 5.55), and DAFO (19.72 ± 4.46). At initial contact children with DAFO presented a significantly increased knee and hip flexion by 4.8° (*p* < 0.016) and 5.3° (*p* = 0.012), respectably, when compared to barefoot walking. No significant difference was found at the ROM in the knee and hip between the AFO and DAFO. There was a significantly higher ground reaction force at the second peak wearing an AFO (0.97 ± 0.06) than when walking barefoot (0.89 ± 0.11). Both the AFO (0.96 ± 0.27) and the DAFO (1.11 ± 0.43) showed a significant improvement in the maximum plantar flexion moment compared to barefoot (0.69 ± 0.25). It was 0.28 Nm/kg higher in the AFO and 0.42 Nm/kg higher in the DAFO. There was no significant difference determined among barefoot, SAFO, and DAFO in all knee and hip power parameters.
Radtka, 1997 [37]	1997	Non-randomized controlled clinical trial (repeated measures design)	10 children with spastic CP (6 diplegic; 4 hemiplegic) (mean age: 6.5 ± 1.86 years)	Spastic diplegia and unilateral CP; community ambulatory with plantigrade foot in standing, excessive plantar flexion during the stance, passive dorsiflexion of 5 degrees or more with knee extended, passive hip extension of 10 degrees or more, passive hamstring muscle length of 60 degrees or more in straight leg raise, mild to moderate spasticity in lower limb; no use of assistive device in ambulation; no orthopedic surgery in the previous year.	10	3 months (2 weeks barefoot + 1 month with AFO + 2 weeks barefoot + 1 month with DAFO)	AFO and DAFO.	Gait analysis data (walking speed; stride length; cadence; range of motion of the trunk, pelvis, hip, knee, and ankle at initial contact and mid-stance).	Contact closing foot switches; optoelectronic system.	There was an increased stride length wearing the AFO (0.97 ± 0.16) and DAFO (0.93 ± 0.13) compared with the barefoot condition (0.82 ± 0.13). The cadence was higher barefoot (148.33 ± 15.73) than with the AFO (140.10 ± 8.79) and DAFO (136.55 ± 10.96). The excessive ankle plantar flexion with no orthoses (8.54 ± 5.61) was over reduced with AFO (−2.62 ± 3.93) and DAFO (−1.66 ± 6.23). There were no differences (*p* < 0.002) at the level in joint motions of the knee, hip, and pelvis at initial contact and mid-stance with AFO or DAFO. The amount of ankle plantar flexion that occurred at initial contact and mid-stance in the interventions with no orthoses was reduced with both AFO and DAFO. No differences were found for the gait variables when comparing the two orthoses (*p* ˂ 0.02).
Radtka, 2005 [36]	2005	Non-randomized controlled clinical trial (repeated measures design)	12 children with spastic diplegic CP (mean age: 7.5 ± 3.83 years)	Spastic diplegia CP; community ambulatory with ankle dorsiflexion to 0 degrees during static standing, excessive ankle plantar flexion of 5 degrees or more during stance in gait, passive ankle dorsiflexion to 5 degrees with knee extended passive hip extension to −10 degrees or less in the Thomas test, passive hamstring length of 50 degrees or more as measured by a straight leg raise; mild spasticity of the triceps surae, hamstrings and quadriceps; no surgical procedures in the past or any other orthopedic surgery during the year prior to the study.	12	3 months (2 weeks barefoot + 1 month with AFO + 2 weeks barefoot + 1 month with HAFO)	SAFO and HAFO.	Gait analysis data (range of motion of the knee and ankle during the stance phase; walking velocity; stride length; cadence; knee and ankle sagittal joint moments and powers during the stance phase).	Optoelectronic system; force plates.	The mean stride length was increased with both SAFO (0.87 ± 0.19) and HAFO (0.90 ± 0.19) when compared to no AFO (0.79 ± 0.19). No significant differences in walking velocity, cadence, and stride length when comparing no AFO, SAFO, and HAFO (*p* ˂ 0.05). At the knee joint there were no findings of significant differences between barefoot, SAFO, or HAFO. When compared to the barefoot condition, at the ankle joint there were significant differences with the AFO and HAFO. The HAFO produced more normal dorsiflexion at the terminal stance phase than the SAFO, and more excessive dorsiflexion during loading phase than barefoot. There were significant differences when comparing no AFO (0.69 ± 0.14), SAFO (0.96 ± 0.22), and HAFO (0.94 ± 0.25) in the peak ankle moments. There was a significant difference in peak ankle moments during the terminal stance phase between barefoot (−1.30 ± 6.59) and SAFO (11.50 ± 4.28) and barefoot and HAFO (16.13 ± 6.17). The mean values were similar between both AFO.
Smith, 2009 [38]	2009	Non-randomized controlled clinical trial plus healthy controls (repeated measures design)	15 children with spastic diplegic CP (mean age: 7.5 ± 2.9 years) + 20 typically developing children (mean age: 10.6 ± 2.8 years)	Spastic diplegia CP; able to walk independently without an assistive device; jump gait pattern; GMFCS level I; no orthopedic surgery in the past 12 months; no botulinum toxin injections in the past 6 months; range of ankle dorsiflexion to at least neutral on static physical examination with the knee extended.	15 + 20	2.5 months (barefoot baseline + 4 weeks with DAFO or HAFO + 2 weeks barefoot + 4 weeks with DAFO or HAFO)	DAFO and HAFO. Barefoot (healthy subjects control group).	Gait analysis data (walking speed; cadence; stride length; range of motion; joint moments; joints powers); functional skills (GMFM scores).	Optoelectronic system; force plates; GMFM.	Significant improvements in gait metrics were seen during brace wear (*p* ≤ 0.05). When compared with barefoot condition, CP children wearing HAFO and DAFO showed a significant increase in stride length (0.98 ± 0.05) and (1.01 ± 0.05) and walking speed (1.09 ± 0.6) and (1.11 ± 0.6). When using HAFO or DAFO there was a significant decrease in normal cadence (*p* ≤ 0.006) compared with the children with CP in barefoot condition. When comparing gait cycles of children with CP and healthy children there was no significant difference in terms of stride length, walking speed, or cadence. At the ankle significant differences between the HAFO or DAFO and the barefoot condition were found during the stance and swing phase (*p* ≤ 0.05). The knee peak flexion during swing was significantly different between the DAFO and barefoot condition (*p* ≤ 0.05). Children with CP using HAFO or DAFO had no significant effect on hip ROM. No significant differences were seen between the two different braces used (*p* ≤ 0.05). The barefoot and braced conditions differed most significantly in terms of ankle kinematics and kinetics (*p* ≤ 0.05). During the terminal stance of pre-swing, the ankle moment was significantly increased for both DAFO (0.98 ± 0.1) and HAFO (1.05 ± 0.1) when compared to the barefoot condition (0.80 ± 0.1). When compared to healthy children, in the barefoot and AFO condition, CP children presented a significant increase in plantar flexor moment during the initial contact (*p* ≤ 0.05). No significant differences in ankle powers were found between DAFO and HAFO.
Zhao, 2013 [30]	2013	Randomized parallel group controlled trial	70 boys and 42 girls with spastic diplegic CP (mean age: 2.69 ± 0.81 years)	Spastic diplegic CP; between 1 and 4 years of age; ability to walk independently, with or without an assistive device; GMFCS levels I-II; able to accept and follow AFO treatment strategy; no unstable seizures; no orthopedic surgery for spasticity within the preceding 6 months; no botulinum toxin injections within the preceding 3 months; without any other diseases that interfered with physical activity, and existence of serious cognitive disabilities.	56 + 56	5 to 8 weeks	Day AFO. Night and day AFO.	Gait analysis data (passive ankle dorsiflexion angle).	Sections D and E of the 66-item GMFM.	No evidence was found that the prolonged wearing time with AFOs leads to increased benefits (*p* ˂ 0.05). The GMFM-66 improvement in the day-night AFO-wearing group was lower than in the day AFO-wearing group rather than higher. AFO day-night use was not more effective than daytime use alone in children with spastic diplegia at GMFCS levels I to II.

Abbreviations: AFO—ankle foot orthoses; CP—cerebral palsy; DAFO—dynamic ankle foot orthoses; GRAFO—ground reaction ankle foot orthoses; GMFCS—Gross Motor Function Classification System; GMFM—Gross Motor Function Measure; HAFO—hinged ankle foot orthoses; ROM—range of motion; SAFO—solid ankle foot orthoses.

**Table 2:** Methodological quality for studies in the review.

Article ID	PEDro Score											Total Score
	Eligibility Criteria *	Random Allocation	Blind Distribution	Similar Prognosis	Blind Subject	Blind Therapist	Blind Assessors	85% Follow-Up	Intention to Treat	Statistical Comparisons	Point of Measure/Measures of Variability	
Bjornson, 2006 [31]	Yes	Yes	No	Yes	No	Yes	Yes	Yes	Yes	Yes	Yes	8/10
Bjornson, 2016 [32]	Yes	Yes	No	Yes	No	No	No	Yes	Yes	Yes	No	5/10
Buckon, 2004 [33]	Yes	Yes	No	Yes	No	No	No	Yes	Yes	Yes	Yes	6/10
Degelean, 2012 [34]	Yes	No	No	No	No	No	No	Yes	Yes	Yes	Yes	4/10
El-Kafy, 2014 [15]	Yes	Yes	Yes	Yes	No	No	Yes	Yes	Yes	Yes	Yes	8/10
Lam, 2005 [35]	Yes	No	No	No	No	No	No	Yes	Yes	Yes	Yes	4/10
Radtka, 1997 [37]	Yes	No	No	Yes	No	No	No	Yes	Yes	Yes	Yes	5/10
Radtka, 2005 [36]	Yes	No	No	Yes	No	No	No	Yes	Yes	Yes	Yes	5/10
Smith, 2009 [38]	Yes	No	No	No	No	No	No	Yes	Yes	Yes	Yes	4/10
Zhao, 2013 [30]	Yes	Yes	Yes	Yes	No	No	Yes	Yes	Yes	Yes	Yes	8/10

* This criterion is cited but not used to compute the total PEDro score.

## Data Availability

All data generated or analyzed during this study are included in this published article.

## Appendix A

### PICO Question Key Words

Population: cerebral palsy; cp; children; children with cerebral palsy; adolescent; diplegia; spastic diplegia.

Intervention: ankle foot orthoses; AFO; orthoses; orthotics; orthosis; ground force reaction orthoses; GRAFO; hinged ankle foot orthoses; HAFO; dynamic ankle foot orthoses; DAFO; solid ankle foot orthoses; SAFO.

Comparison: (none).

Outcome: gait; kinematics; kinetics; walking; functionality; functional activities; gait pattern; gait velocity; trunk sway; maximum knee extension; maximum hip extension; ankle; knee; hip; range of motion; ROM; gross motor function; GMFM; walking speed; stride length; energy expenditure.

Search Strategies (MeSh terms; word truncation; relevance of key words).

1. “cerebral palsy” [mh]
2. child *[mh]
3. adolescent
4. #1–#3
5. “sagittal gait patterns”
6. “spastic diplegia”
7. “true equinus”
8. “jump gait”
9. “apparent equinus”
10. “crouch gait”
11. “asymmetric gait”
12. #5–#11
13. “ankle foot orthoses”
14. AFO
15. “orthotic devices” [mh]
16. “foot orthoses” [mh]
17. splints [mh]
18. #12–#17
19. gait [mh]
20. walking [mh]
21. kinematics [mh]
22. kinetics [mh]
23. “spatiotemporal analysis”
24. functionality
25. “functional activities”
26. ICF
27. “gross motor function measure”
28. #19–#27
29. “randomised controlled trial” [pt]
30. “controlled clinical trial” [pt]
31. “clinical trial” [pt]
32. “comparative study” [pt]
33. #29–#32
34. #1–#3 AND #5–#11 AND #12–#17 AND #19–#27 AND #29–#32

Search Questions used in different data sources

#1:

((“cerebral palsy” [mesh] OR child* [mesh] OR adolescent [mesh]) AND (“sagittal gait patterns” OR “spastic diplegia” OR “true equinus” OR “jump gait” OR “apparent equinus” OR “crouch gait” OR “asymmetric gait”) AND (“ankle foot orthoses” OR AFO OR “orthotic devices” [mesh] OR “foot orthoses” [mesh] OR splints [mesh]) AND (gait [mesh] OR walking [mesh] OR kinematics [mesh] OR kinetics [mesh] OR “spatiotemporal analysis” OR functionality OR “functional activities” OR ICF OR “gross motor function measure”) AND (“randomized controlled trial” [pt] OR “controlled clinical trial” [pt] OR “clinical trial” [pt] OR “comparative study” [pt]))

#2

(“cerebral palsy” OR child* OR adolescent OR youth) AND (“sagittal gait patterns” OR “spastic diplegia” OR “true equinus” OR “jump gait” OR “apparent equinus” OR “crouch gait” OR “asymmetric gait”) AND (“ankle foot orthoses” OR AFO OR “orthotic device*” OR orthos* OR “foot orthos*” OR splint*) AND (gait OR “walking speed” OR walking OR ambulation OR kinematics OR kinetics OR biomechanical OR “spatiotemporal analysis” OR functionality OR “functional activities” OR ICF OR “gross motor function measure”) AND (“randomized controlled trial” OR “controlled clinical trial” OR “clinical trial” OR “comparative study”)

#3:

(“cerebral palsy”) AND (“sagittal gait patterns”) OR (“spastic diplegia”) AND (“ankle foot orthoses”) OR (gait) OR (kinematics) OR (kinetics)

**Table A1:** Search Results.

Date	Source	Search Question	Nº of Results	Notes
13 January 2020	Pubmed	#1	14	
27 January 2020	Scopus	#2	363	
27 January 2020	Isi Web of Science	#1	8	No filter
27 January 2020	Scielo	#3	17	No filter
27 January 2020	Cochrane	#1	6	
